# Real-time intraoperative functioning lumen imaging probe during endoscopic per-oral pyloromyotomy (pop)

**DOI:** 10.1007/s00464-020-08237-5

**Published:** 2021-01-11

**Authors:** Alisan Fathalizadeh, Michael Klingler, Joshua Landreneau, Matthew Allemang, John Rodriguez, Jeffrey Ponsky, Kevin El-Hayek

**Affiliations:** 1 Digestive Disease and Surgery Institute, Cleveland Clinic, Cleveland, OH USA; 2 Division of General Surgery, MetroHealth System, Cleveland, OH USA; 3 Case Western Reserve University School of Medicine, Cleveland, OH USA

**Keywords:** FLIP, POP, Gastroparesis, G-POEM, Functional lumen imaging probe, Pyloromyotomy

## Abstract

**Background:**

Endoscopic per-oral pyloromyotomy (POP) has emerged as a safe and effective first line option in medically refractory gastroparesis. Determining the appropriate extent of the pyloromyotomy continues to present a challenge as there are no standardized tools for measuring changes in pyloric distensibility during the procedure. The objective of this study was to evaluate the utility of using impedance planimetry with endoscopic functional luminal imaging probe (FLIP) to measure changes in pyloric distensibility after POP, and to compare these changes with improvement in symptoms and objective gastric emptying.

**Methods:**

Patients with medically refractory gastroparesis underwent POP with FLIP measurements of the pylorus (EndoFLIP®, Medtronic, Fridley MN). FLIP measurements, as well as changes in symptoms measured by the validated gastroparesis cardinal symptom index (GCSI) and scintigraphic gastric emptying studies (GES), were evaluated before and after POP.

**Results:**

A total of 14 patients underwent measurement with FLIP during POP, 12 of whom had pre- and post-POP measurements. Mean pyloric diameter increased by 1.4 mm, from 13.9 mm to 15.3 mm (*p* = 0.0012). Mean distensibility index increased from 6.2 mm^2^/mmHg to 9.1 mm^2^/mmHg (p = 0.0074). Successful division of the pylorus was achieved in 100% of patients with a mean operative time of 36 min and no perioperative complications. The mean length of stay was 0.7 days (0–3 days). Post-POP mean GCSI score improved from 2.97 to 2.28 at a mean follow-up time of 27 days (*p* < 0.001). Objective improvement in gastric emptying was observed in 80% of patients with scintigraphic GES, with mean four-hour retention decreasing from 46.3% to 32.4% (p < 0.007).

**Conclusions:**

FLIP is a safe and feasible tool to provide objective measurements during POP. Larger cohorts with longer follow-up are required to determine if measured improvements in pyloric diameter and distensibility are predictive of sustained improvements in GCSI and GES.

Gastroparesis is a functional disorder of the stomach defined as delayed gastric emptying without mechanical obstruction. Gastroparesis is typically categorized as idiopathic, diabetic, or post-surgical. Among the various etiologies, sustained, high-amplitude contration of the gastric pylorus, “pylorospasm,” has been considered a potential causative mechanism of gastroparesis [1]. This observation has led to the development of pylorus-directed therapies such as endoscopic dilations and botulinum toxin injections to relax the pyloric sphincter to provide temporary relief of gastroparesis symptoms [2]. Surgical options, including gastric electrical stimulation, pyloroplasty, or subtotal gastrectomy, offer more sustained improvement at the expense of adding significant surgical morbidity [3–5].

Per-oral pyloromyotomy (POP), also known as gastric per-oral endoscopic myotomy (G-POEM), is a safe and effective endoscopic approach for gastroparesis with similar functional outcomes, less morbidity, and decreased length of stay when compared to surgical pyloroplasty [3]. Improvements in both gastroparesis symptoms and objective gastric emptying are achieved in roughly 70% of patients after POP [6]. While these data are promising, procedural success and positive outcomes following POP are dependent on complete division of the pyloric sphincter. There are currently no standardized methods for objectively determining changes in the pyloric distensibility during the procedure, which would help to assure the surgeon that a complete pyloromyotomy has been performed.

Endoscopic functional luminal imaging probe (FLIP), EndoFLIP® (Medtronic, Fridley MN) technology, involves a balloon with 16 sensors mounted on a thin catheter that can be deployed and maneuvered endoscopically. FLIP is indicated to measure the intraluminal pressure, diameter, and distensibility index (DI) of gastrointestinal sphincters. Use of FLIP has demonstrated an increase in esophagogastric junction diameter and distensibility after completion of per-oral endoscopic myotomy (POEM) for achalasia. These results have been correlated with clinical response after POEM. Several studies have also shown the utility of FLIP measurements in the assessment of the physiological properties of the pylorus [7–11].

The objective of this study was to determine the safety and feasibility of real-time, intraoperative FLIP in patients undergoing POP. These data could potentially help determine if increased pyloric distensibility as measured by FLIP correlates with improvement in GCSI and GES.

## Materials and methods

### Study population

This was a retrospective study performed at a single academic referral center. IRB approval was obtained through the Cleveland Clinic system. Consent was obtained prior to performing the POP and FLIP procedures. All patients with medically refractory gastroparesis who underwent POP with FLIP from April 2019 to June 2019 were included. Refractory gastroparesis was defined as symptoms refractory to standard medical therapy which includes diet, life style modification, anti-emetics, and pro-motility agents [1]. Patients with gastroparesis underwent work-up in a standardized manner consisting of preoperative assessment with symptom severity scoring via the validated Gastroparesis Cardinal Symptom Index (GCSI) and 4-h objective emptying measured by nuclear gastric emptying scintigraphy (GES). GCSI scores are divided into three subcategories: post-prandial fullness/early satiety (4 items); nausea/vomiting (3 items), and bloating (2 items) whereby higher scores in each subcategory are correlated with worse symptoms [12]. Patient demographics, etiology of gastroparesis, procedure details, complications, pre- and post-GCSI data were collected from the electronic medical record. Post-procedure GCSI scores were initially obtained at routine 1-month clinic visits. Follow-up nuclear GES was performed 3 months post procedure. Clinical outcome was measured by an improvement in the GCSI and GES after the POP procedure [1].

### FLIP and POP procedure

All procedures were performed by surgical endoscopists experienced in performing POP, with patients under general anesthesia with endotracheal intubation. The procedure is typically performed as an outpatient procedure at our institution. Rarely, a patient may be admitted overnight for observation. Patients are maintained on a full liquid diet followed by soft diet for two weeks which is advanced as tolerated. Patients are educated to eat small portions 4–5 times daily.

EndoFLIP® technology was employed to provide an objective measurement of pyloric diameter and distensibility immediately before and after POP. The device employs impedance planimetry technology to measure cross-sectional area, diameter, and distensibility within a balloon-tipped catheter. The catheter contains a series of small paired electrodes. A small electrical current is passed through the balloon filled with conductive fluid to the paired electrodes. This voltage gradient between electrodes is used to measure the diameter and cross-sectional area of the lumen, as there is an inverse relationship between cross-sectional area and the voltage gradient at the midpoint between electrodes. The change in cross-sectional area along with the change in pressure measured via balloon catheter is used to calculate the distensibility index [13]. The EndoFLIP® software uses these data to generate a topographic image of the pressure dynamics, which can be viewed by the surgeon in real time.

### FLIP procedure

**Fig. 1:**
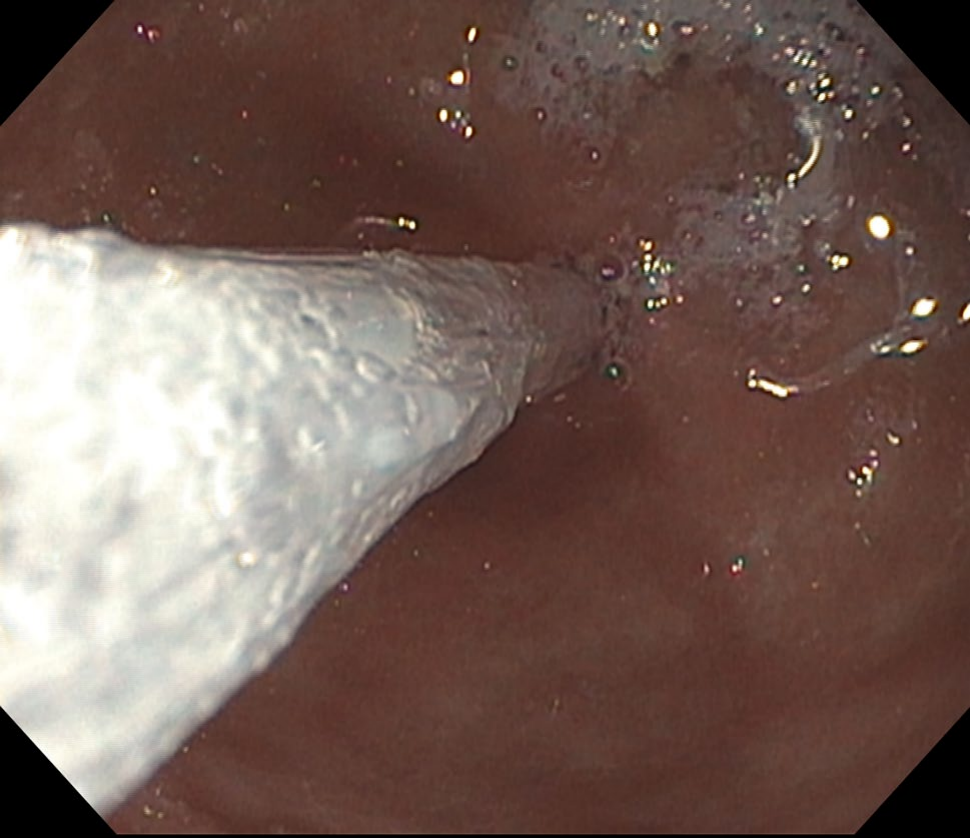
Passage of the balloon-tipped catheter through the pylorus

The FLIP catheter may be placed using an endoscopic grasper or in a through-the-scope method. A standard upper gastroscope (GIF-HQ190, Olympus, Tokyo, Japan) is used in the endoscopic grasper approach, the FLIP catheter is passed through the patients mouth into the stomach, and a grasper, which is passed though the endoscope, is used to manually advance the FLIP probe into the pylorus (Fig.  1).

In the through-the-scope method, the catheter is passed through the channel of a therapeutic videogastroscope with a 6 mm working channel (GIF-XTQ160, Olympus, Tokyo, Japan) into the pylorus. Endoscopes with a larger working channel may also be employed. For ease of catheter placement and manipulation, we opted to predominantly use this technique.

**Fig. 2:**
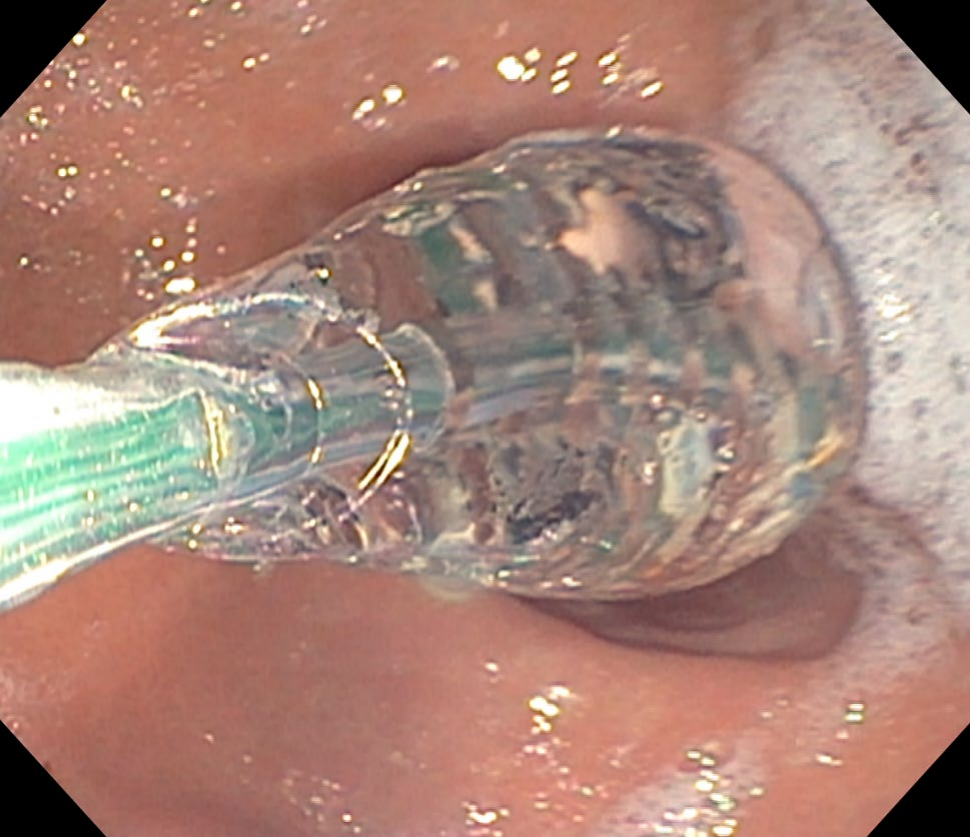
Starting inflation of the balloon-tipped catheter while traversing the pylorus

**Fig. 3:**
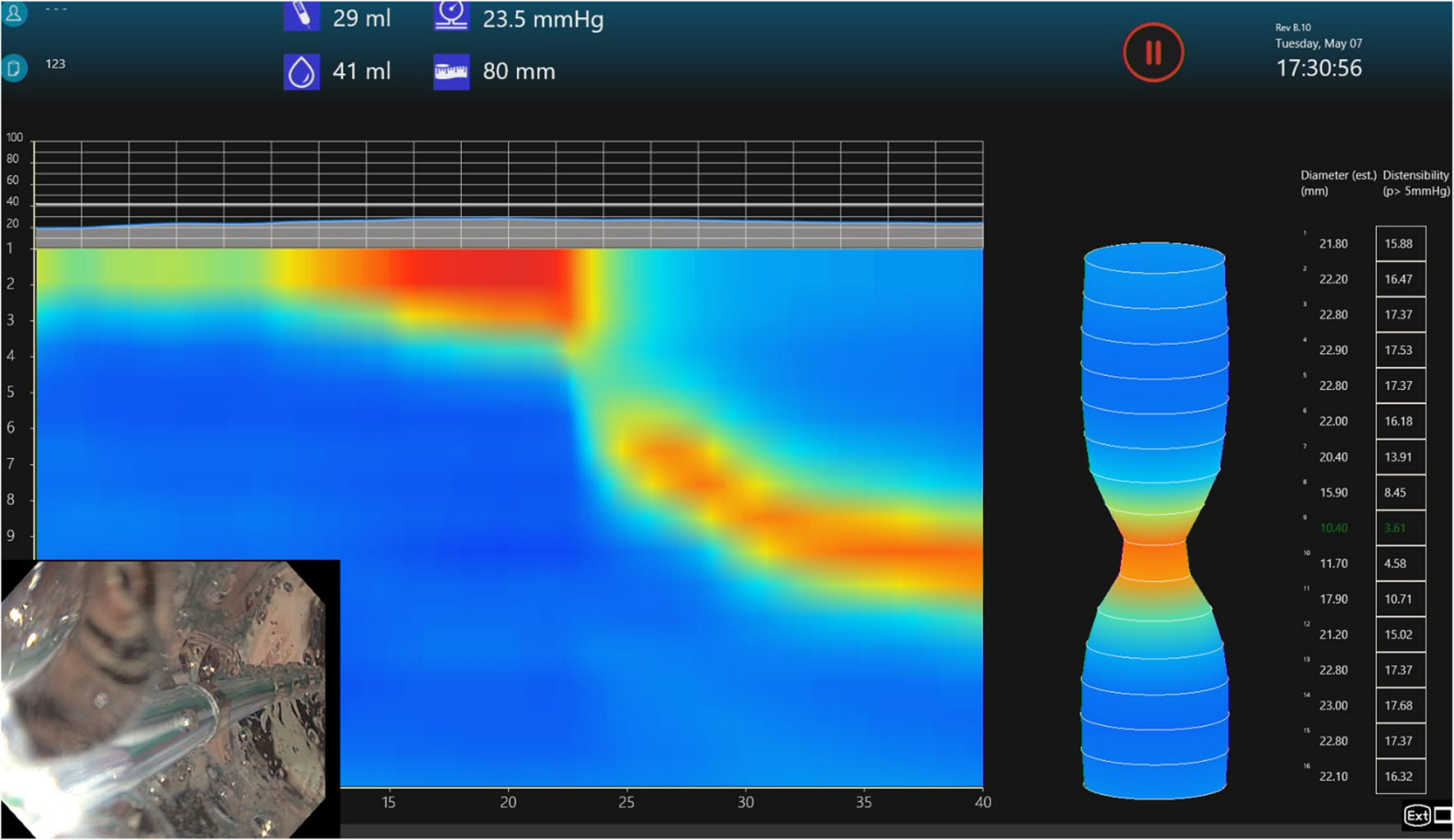
Pre-POP topographic image generated by FLIP software demonstrating the pressure dynamics within the balloon catheter while the balloon is inflated. D-10 mm, DI-3.61 mm^2^/mmHg

A 240 cm catheter with an 8-cm-long 3-mm balloon tip (EF-325 N) was used in all cases. A 16 cm balloon tip length (EF-322 N) is also available; however, we chose the 8 cm balloon due to the small size of a pylorus and to limit variability of devices used. The FLIP balloon was stabilized and inflated to a fixed volume of 40 cc in all cases, which has been shown to be the optimal volume for evaluating pyloric sphincter contour [8] (Fig. 2). After waiting at this fixed volume for thirty seconds, the median cross-sectional area/diameter and distensibility were measured at the most narrow part of the pylorus as represented on the topographic map (Fig. 3).

### POP procedure

**Fig. 4:**
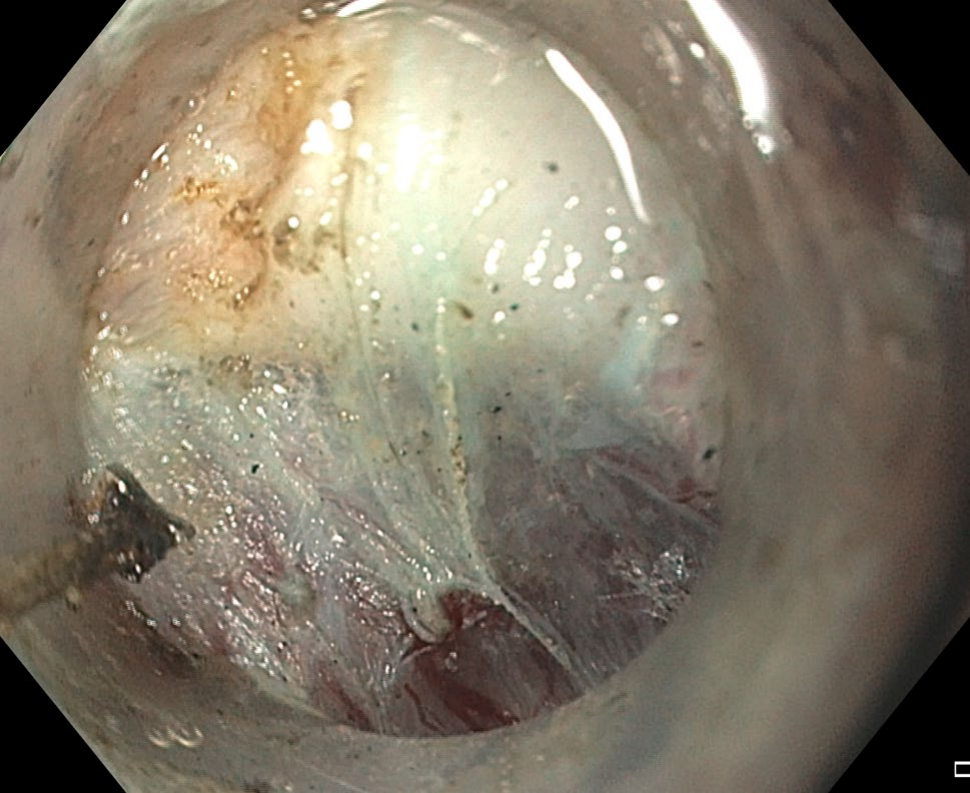
Pylorus muscle visualized as a thickened white band prior to performing a pyloromyotomy

**Fig. 5:**
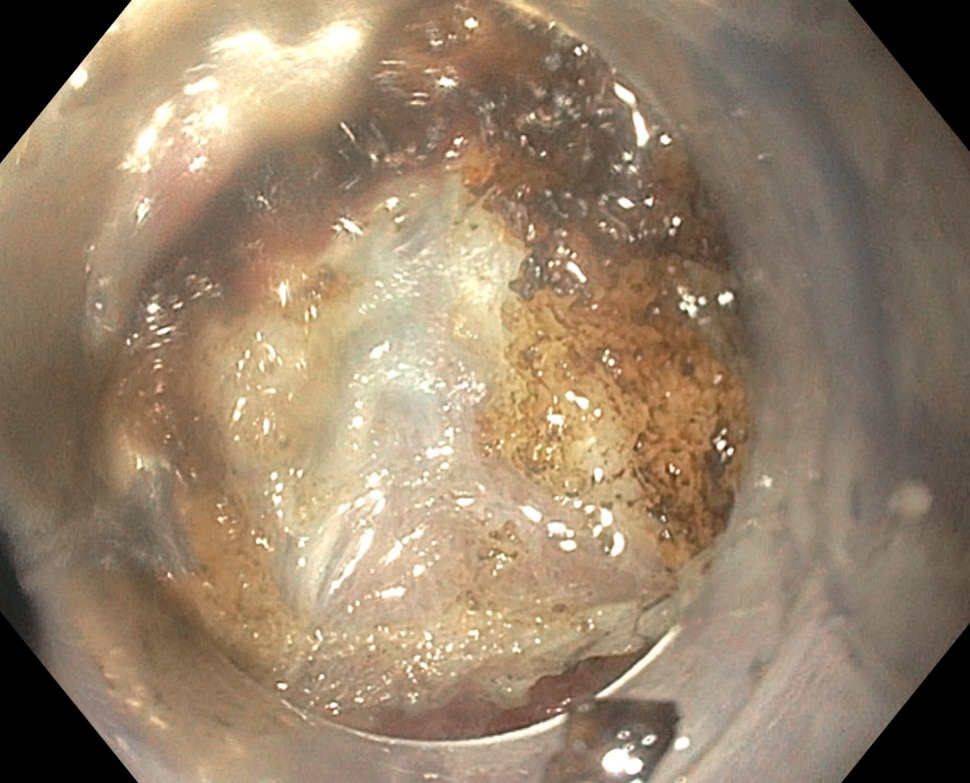
Pylorus muscle divided after a POP procedure

**Fig. 6:**
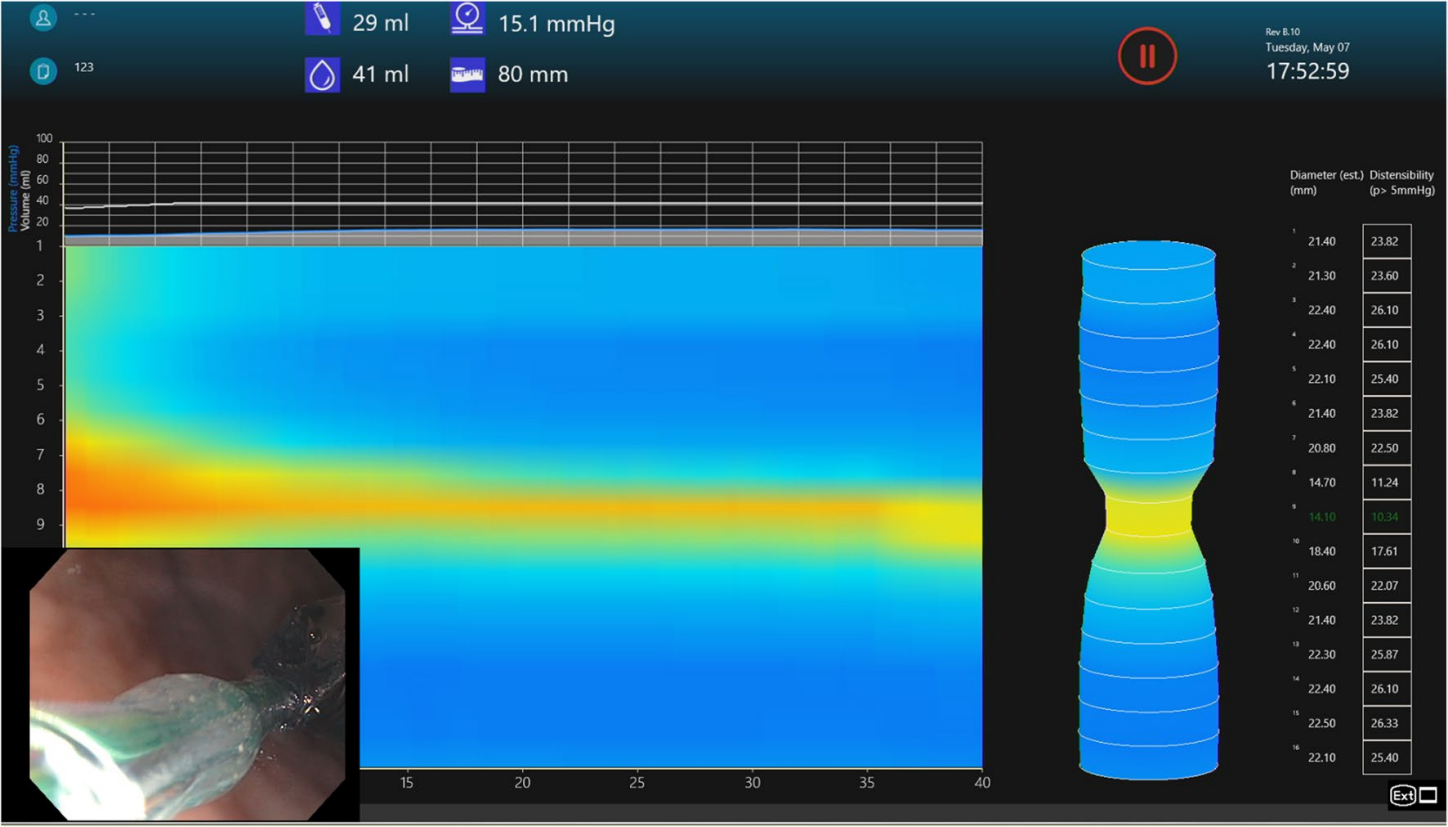
Post-POP FLIP topographic image generated by FLIP software demonstrating the pressure dynamics within the balloon catheter while the balloon is inflated. D-14 mm, DI-10.34 mm^2^/mmHg

A standard upper gastroscope (GIF-HQ190, Olympus, Tokyo, Japan) with an attachable silicone cap (Barrx™ RFA Cleaning Cap, Medtronic, Fridley MN) was used to perform POP. After baseline FLIP measurements, POP was performed in a consistent stepwise manner for each patient: (1) diagnostic endoscopy is performed without passing the scope through the pylorus to reduce tissue trauma, (2) creation of a submucosal bleb 2 cm proximal to the pylorus on the lesser curve, followed by a 1.5–2 cm transverse mucosotomy, (3) submucosal tunnel creation (Fig. 4), (4) pyloromyotomy (Fig. 5), and (5) mucosotomy closure with clips. The technical aspects of the POP have been previously described [14]. Technical success of the POP procedure was defined by successful completion of all of the aforementioned steps. Immediately following the pyloromyotomy, FLIP measurements of diameter and distensibility were again performed in an identical fashion to the initial assessment (Fig. 6). In at least one patient, the FLIP data before and after the POP did not demonstrate a substantial change and therefore further myotomy was performed and the DI did demonstrate an appropriate improvement.

## Statistical analysis

Data were described using means and standard deviations or medians with interquartile ranges for continuous variables and counts and percentages for categorical variables. Comparisons of pre and post pyloric diameter and distensibility measurements, as well as pre and post GCSI and GES, were done by paired *t*-tests. All analyses were performed on a complete-case basis. All tests were two-tailed and performed at a significant level of 0.05. R software 3.5.0 (04/23/2018, Vienna, Austria) was used for all analyses.

## Results

Fourteen patients with gastroparesis underwent POP with FLIP during the study period. Median age was 41 (IQR 16–67). Gastroparesis etiology was as follows: diabetic gastroparesis (6), idiopathic (7), and post-surgical (1). The median pre-procedure GCSI score was 3.1 with bloating as the most prominent reported symptom. Median post-procedure GCSI score at one month was 2.2 which demonstrated clinical improvement. Mean GCSI score improved from 2.97 to 2.28 after POP at a mean follow-up of 1 month.

The median pre-POP pyloric diameter was 13.2 mm. On average, patients demonstrated a significant increase in pyloric diameter of 1.4 mm (*p* = 0.0012) from a mean diameter of 13.9 mm to 15.3 mm at the time of the POP procedure. The average distensibility index (DI) significantly increased by 2.9 mm^2^/mmHg, from 6.2 mm^2^/mmHg to 9.1 mm^2^/mmHg at the time of POP (*p* = 0.007) [Table 1]. Technical success for POP was achieved in 14/14 (100%) of patients. Technical success for FLIP procedure was achieved in 12/14 (86%), where post-POP measurements were unable to be obtained due to catheter malfunction. Mean operative time was 36 min and mean length of hospital stay was 0.7 days (0–3 days). There were no perioperative complications. Follow-up scintigraphic gastric emptying studies were available in five patients. Overall, 4 out of 5 patients (80%) had improvement in scintigraphic gastric emptying compared to preoperative assessments. One patient (20%) had worsening GES despite improvement in gastroparesis symptoms as measured by GCSI. The follow-up gastric emptying study results after the POP procedure demonstrated an improvement from 46.3% to 32.4% (*p* < 0.007) [Table 2] [15].

**Table 1:**
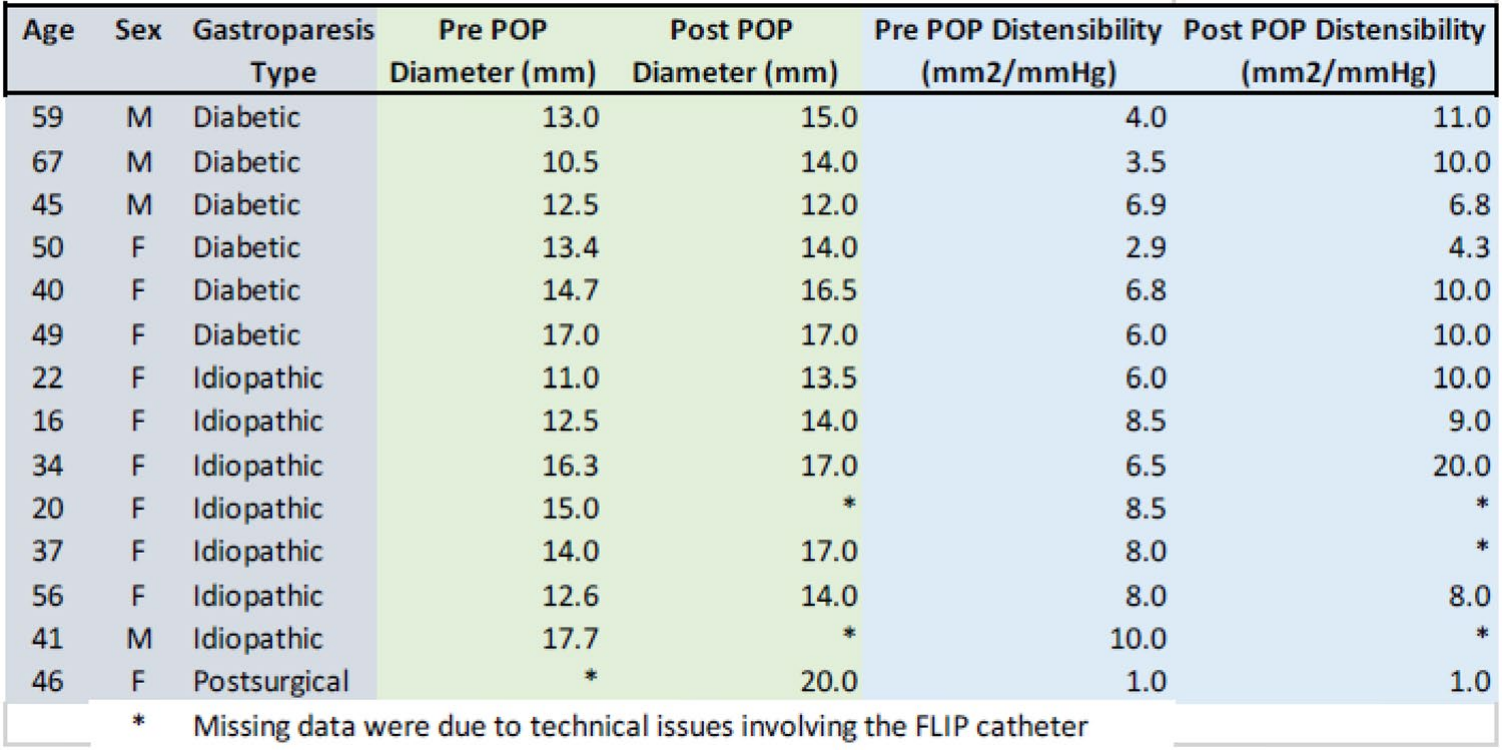
Demographics and comparison of pre- and post-POP diameter and distensibility

*Missing data were due to technical issues involving the FLIP catheter

**Table 2:**
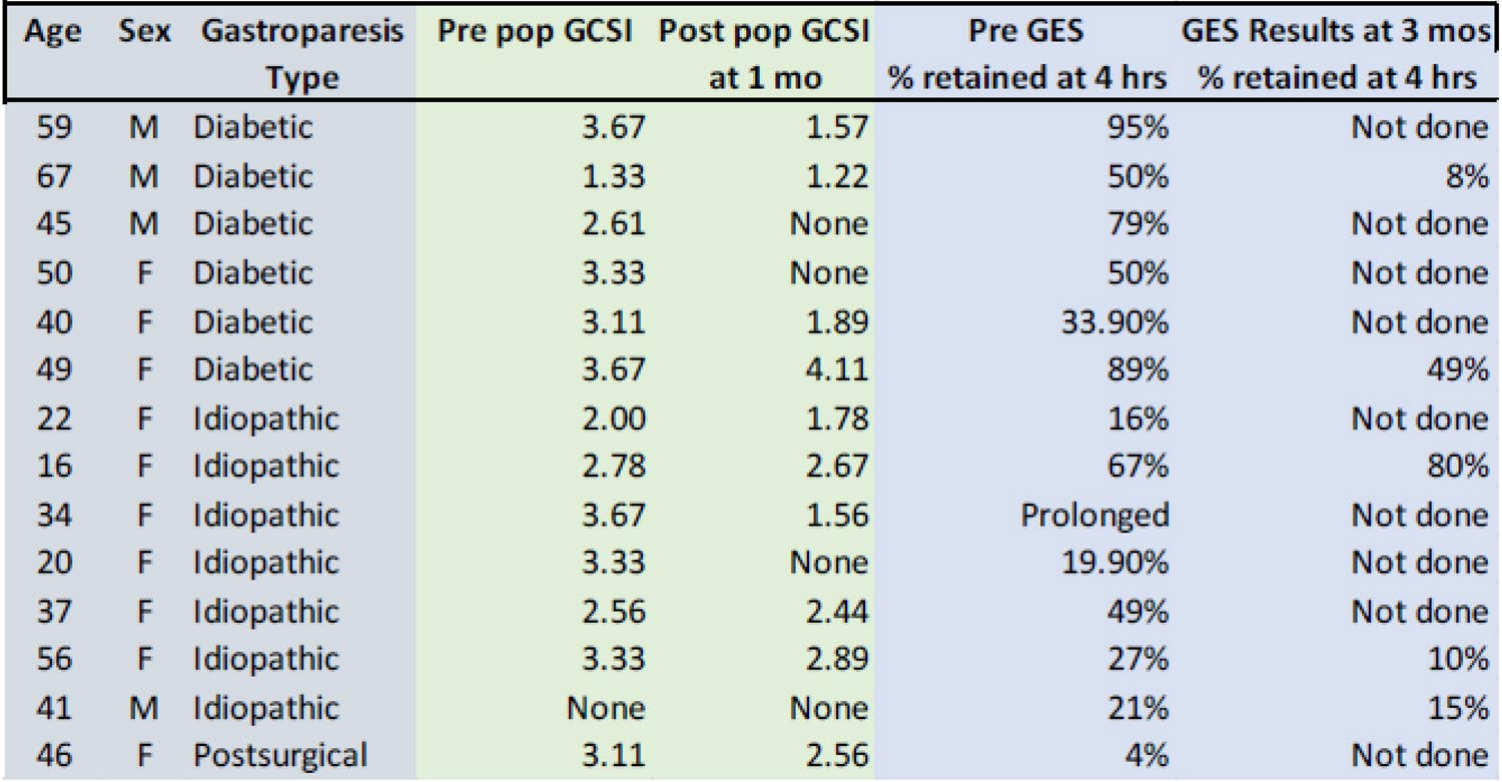
Demographics and Pre- and Post-POP GCSI and GES

## Discussion

Prior to the use of FLIP, improvement in the pyloric diameter after POP was largely based on visual cues and the ease of passage of the endoscope through the pylorus after pyloromyotomy. Real-time measurements of distensibility and pressure using FLIP, as represented by a topographical image displayed during the procedure, give instant feedback to the endoscopist that can be used during the procedure to evaluate the extent of the pyloromyotomy and to make appropriate adjustments at that time.

The results from the present study support that FLIP is safe and feasible, and a significant improvement in pyloric diameter and distensibility was seen in patients who underwent POP for gastroparesis of varying etiologies. This is consistent with previously reported use of this technology following POP or pyloric botulinum toxin injection [16]. Patients also experienced significant improvements in both gastroparesis symptoms and objective gastric emptying times, although the cohort size was too small to evaluate the relationship between improvement in distensibility and GCSI/GES measurements. We used a standardized approach in measuring distensibility via FLIP, which was done immediately before and after pyloromyotomy. Using a non-standardized approach for FLIP measurements, which were taken intraoperatively and up to 3 months post POP, Vosoughi and colleagues were able to demonstrate a correlation between higher distensibility indices/cross-sectional areas and clinical success after POP in a study of patients with gastroparesis. Additionally, post-POP cross-sectional area was predictive of clinical improvement with a high specificity (91%) and reasonable sensitivity (71%) [1]. A case series of four patients with pre and post POP FLIP procedures showed increased cross-sectional area was associated with clinical response at 3 months [1]. Finally, these investigators demonstrated that immediate post-POP evaluation of cross-sectional area and distensibility index was a better predictor of clinical success compared to measurements at 3 months. Performing the FLIP data at 3 months is reasonable; however, it requires an additional endoscopic procedure which is not part of our standard practice. In addition to being a better predictor of clinical success, immediate post-POP measurements with FLIP allows for real-time evaluation of extent of myotomy such that further myotomy can be performed and outcomes immediately evaluated. This pilot study aimed to assess safety and efficacy of this technology but real-time use of the FLIP technology can be used in the future to guide further myotomy as appropriate. It is likely the delta change in values of diameter and DI will be more valuable than absolute values with a target of delta change > 2 or DI greater than 10 if technically possible.

Routine use of FLIP during POP could help to identify which patients may have the greatest clinical improvement based on their baseline pyloric measurements. Previous studies have demonstrated that higher pre-POP distensibility indices were associated with 1-year clinical success and improvement in abdominal pain [1, 17]. Routine use of FLIP may help to establish a goal range of improvement in pyloric distensibility, where measurements below this threshold could indicate a need to perform a more extensive pyloromyotomy. In at least one patient the results of the FLIP technology were used in real time to perform a more extensive myotomy which demonstrated noticeable change in the distensibility index. A change in the DI appears to be more valuable than the actual recorded value. The degree of improvement in distensibility may also predict clinical response, which warrants further study with larger cohorts. This may be the case, as low pyloric compliance has been shown to correlate with worse gastroparesis symptoms [11]. In our patient group, four out of five patients who had a follow-up gastric emptying scintigraphy demonstrated objective improvement in gastric emptying times which is consistent with previous studies demonstrating an improvement rate of 78.3% [15]. The ability to see measurements using the FLIP technology in real time and to understand the correlation to clinical outcomes will allow this technology to be used to ensure the adequacy of myotomy and to make appropriate changes at the time of the procedure.

There are several technical considerations regarding endoscopic placement of the FLIP catheter at the pylorus. First, the catheter relies on the communication of a series of electrodes which can be compromised by manipulation during the procedure. Post-POP FLIP measurements were not able to be obtained in two patients, likely due to disruption of the electrodes that occurred during placement into the stomach. Additionally, the catheter tends to lose tensile strength with manipulation, making positioning more difficult during post-POP measurements. This challenge was mitigated by eventually switching to a therapeutic videogastroscope with a 6 mm working channel to reduce manual manipulation of the catheter. Alternatively, if this is not available, a raptor forceps may be used to drag the catheter alongside the scope into the duodenum but this is more technically challenging and causes greater catheter manipulation. Second, the device employs a balloon at the tip of the catheter with a preselected volume of 40 mL, which is thought to be optimal for the pylorus [18]. The pylorus can be quite narrow in some gastroparesis patients and inflating the balloon within the pyloric channel appeared to dilate the muscle in some cases, leaving a visibly larger channel when the balloon was removed. The pre-POP diameter and distensibility may be underestimated if the balloon is causing a degree of pyloric dilation or relaxation, and attempts at using a smaller volume of 30 cc did not produce similar quality topographic images of the pylorus [19]. Additionally, there is a theoretical risk of pyloric trauma which may obscure tissue planes making POP more challenging, although we did not experience this in our series. Ultimately, the risk of catheter malfunction is a real possibility. To mitigate this risk one may first trial the technology on a model or use it while supervised by a proctor or company representative. We identified a learning curve that improved considerably after five uses of the catheter. We recommend using the technology at least 5 times to lessen the learning curve and consider being proctored with initial uses. Additionally, it is preferable to use a therapeutic endoscope which will allow passage of the FLIP catheter directly through the channel into the duodenum. If this is unavailable, a raptor grasper may be used but requires grabbing the tip of the catheter and dragging it alongside the endoscope into the duodenum. In our experience this was more likely to cause catheter malfunction than with the therapeutic endoscope. Also, we recommend obtaining the FLIP measurements prior to placing clips which may cause damage to the balloon on the catheter.

There are several limitations to this study. First, the study was conducted at a single, tertiary care institution with very high volume of POP procedures, which may limit generalizability. Second, data were gathered prospectively although the analysis was completed retrospectively. Lastly, the small sample size resulted in lack of statistical power to perform an in-depth analysis of subgroups of gastroparesis, including comparisons of improvement in pyloric distensibility with improvements seen in GCSI scores and gastric emptying. Additionally, gastroparesis patients may use different medications to help with symptomatic relief including but not limited to anti-emetics and pro-motility agents. The use of these medications prior to and after the POP procedure has been shown to decrease demonstrating symptomatic release in previously published studies; however, the use of these medications was not tracked in this study evaluating EndoFLIP at the time of POP but would be beneficial to evaluate in future studies. Despite these limitations, this series described a real-time intraoperative FLIP protocol to evaluate improvements in pyloric diameter and distention in patients undergoing POP.

## Conclusion

FLIP is a novel technology that may be used to provide objective measures of pyloric physiology in real time before and after POP. Data from our study suggest improvements in pyloric distensibility occur immediately after POP. Larger studies with long-term follow-up are needed to elucidate correlations between an increase in pyloric diameter/distensibility and the improvement in symptoms and gastric emptying times. Such data may be used in real time to ensure adequate pyloromyotomy and to predict the response to POP in patients with medically refractory gastroparesis.
